# The Insulin Receptor Substrate 1 (Irs1) in Intestinal Epithelial Differentiation and in Colorectal Cancer

**DOI:** 10.1371/journal.pone.0036190

**Published:** 2012-04-27

**Authors:** Diana L. Esposito, Federica Aru, Rossano Lattanzio, Annalisa Morgano, Michela Abbondanza, Reza Malekzadeh, Faraz Bishehsari, Rosa Valanzano, Antonio Russo, Mauro Piantelli, Antonio Moschetta, Lavinia Vittoria Lotti, Renato Mariani-Costantini

**Affiliations:** 1 Unit of General Pathology, Aging Research Center, G. d'Annunzio University Foundation, Chieti, Italy; 2 Department of Medical, Oral and Biotechnological Sciences, G. d'Annunzio University, Chieti, Italy; 3 Laboratory of Lipid Metabolism and Cancer, Department of Translational Pharmacology, Consorzio Mario Negri Sud, Santa Maria Imbaro, Chieti, Italy; 4 Department of Experimental Medicine, University La Sapienza, Rome, Italy; 5 Digestive Disease Research Center, Shariati Hospital, University of Tehran, Tehran, Iran; 6 Department of Clinical Physiopathology, University of Florence, Florence, Italy; 7 Department of Surgical and Oncological Sciences, University of Palermo, Palermo, Italy; Texas A&M University, United States of America

## Abstract

Colorectal cancer (CRC) is associated with lifestyle factors that affect insulin/IGF signaling, of which the insulin receptor substrate 1 (IRS1) is a key transducer. We investigated expression, localization and pathologic correlations of IRS1 in cancer-uninvolved colonic epithelium, primary CRCs with paired liver metastases and *in vitro* polarizing Caco2 and HT29 cells. IRS1 mRNA and protein resulted higher, relative to paired mucosa, in adenomas of familial adenomatous polyposis patients and in CRCs that overexpressed *c-MYC*, ß-catenin, InsRß, and IGF1R. Analysis of IRS1 immunostaining in 24 cases of primary CRC with paired colonic epithelium and hepatic metastasis showed that staining intensity was significantly higher in metastases relative to both primary CRC (*P*<0.01) and colonic epithelium (*P*<0.01). Primary and metastatic CRCs, compared to colonic epithelium, contained significantly higher numbers of IRS1-positive cells (*P* = 0.013 and *P* = 0.014, respectively). Pathologic correlations in 163 primary CRCs revealed that diffuse IRS1 staining was associated with tumors combining differentiated phenotype and aggressive markers (high Ki67, p53, and ß-catenin). In Caco 2 IRS1 and InsR were maximally expressed after polarization, while IGF1R was highest in pre-polarized cells. No nuclear IRS1 was detected, while, with polarization, phosphorylated IRS1 (pIRS1) shifted from the lateral to the apical plasma membrane and was expressed in surface cells only. In HT29, that carry mutations constitutively activating survival signaling, IRS1 and IGF1R decreased with polarization, while pIRS1 localized in nuclear spots throughout the course. Overall, these data provide evidence that IRS1 is modulated according to CRC differentiation, and support a role of IRS1 in CRC progression and liver metastatization.

## Introduction

Colorectal cancer (CRC) has been linked to lifestyle risk factors, most notably diets based on energy-dense foods and low physical activity [1]–[3]. Epidemiological and experimental evidences indicate that the hormone insulin and the insulin-like growth factors (IGFs) 1 and 2 play key role(s) in mediating the complex effect(s) of diet and exercise on CRC risk [4]–[10]. Over-expression of the insulin receptor (InsR) and of the closely related IGF1 receptor (IGF1R) is critical for insulin/IGF system over-activation in cancer [4], [7], [9]. Intestinal epithelium, that possesses one of the highest renewal rates among human tissues, expresses both the InsR and the IGF1R, and the levels of these receptors are higher in CRC relative to colonic mucosa [4], [7], [8], [11].

Intestinal epithelial differentiation is regulated by several pathways, particularly ß-catenin-dependent WNT signaling [12], [13]. Most CRCs appear to initiate after inactivating mutations in the adenomatous polyposis coli (*APC*) gene, encoding a central component of the cytosolic multi-protein complex that controls ß-catenin degradation [14]–[16]. *APC*-mutated cells show high cytoplasmic and nuclear ß-catenin; the latter, after binding to TCF/LEF transcription factors, forms complexes that, by switching on several cancer-related genes, impose a proliferative crypt progenitor phenotype (reviewed at http://www.stanford.edu/~rnusse/wntwindow.html) [17].

Interestingly, recent evidences link the ß-catenin and the insulin/IGFs signaling pathways. In fact, *IRS1*, encoding one of the two major insulin receptor substrates (IRS1 and IRS2), that integrate signaling from the InsR, IGF1R and other cytokine and growth factor receptors [18], is highly upregulated in cells with exogenously-induced or constitutive ß-catenin signaling [19]. This seems to depend on direct regulation of *IRS1* by TCF/LEF-ß-catenin complexes. Furthermore, IRS1 is necessary for transformation in cells that ectopically express oncogenic ß-catenin and for maintainance of the neoplastic phenotype in *APC*-mutated cells [19]. These findings are consistent with earlier evidence that ectopic IRS1 promotes transformation, while a dominant-negative *IRS1* mutant acts as a tumor suppressor [20]. Furthermore, in the *Apc*(*Min*/+) mouse model, intestinal tumorigenesis is attenuated by *irs1* knock-out [21].

In the present study we investigated the expression, localization and clinicopathologic correlations of IRS1 *ex vivo*, in cancer-uninvolved human colonic epithelium, primary CRCs and paired liver metastases, and *in vitro*, in two CRC cell models capable of spontaneous *in vitro* polarization, Caco2 and HT29 [22], [23].

## Materials and Methods

### Patients and specimens

A formalin-fixed, paraffin-embedded (FFPE) series of 24 primary CRCs with paired cancer-uninvolved colonic mucosa and synchronous liver metastasis was retrospectively identified at the Department of Surgical and Oncological Sciences, University of Palermo, Palermo, Italy. For this series standard whole sections were used for immunohistochemistry (IHC). An additional FFPE series, consisting only of primary CRCs, provided by the Digestive Disease Research Center (DDRC), Tehran University of Medical Sciences, Tehran, Iran, included 163 of the 205 CRC cases described in Bishehsari et al. and in Mahdavinia et al. [24], [25], selected based on tissue availability. These CRCs had been previously characterized for microsatellite instability (MSI) status and *p53* and *KRAS* mutations [24], [25]. Clinico-pathological data, including age and sex, tumor size, stage and grade, were available for all 163 patients. No follow-up and survival data were available. Tissue microarrays (TMAs) were constructed by extracting histologically-confirmed CRC cores from donor blocks with a Beecher MTA 2-mm Punch Set (Beecher Instruments, Sun Prairie, WI, USA). The cores were re-embedded into gridded paraffin blocks and standard TMA sections were used for IHC.

Samples of tumor and paired colonic mucosa, snap-frozen or rapidly fixed in RNAlater (Ambion, Applied Biosystems, Foster City, CA), were collected at the Department of Clinical Physiopathology, University of Florence, Florence, Italy, from 8 unselected CRC cases and 2 familial adenomatous polyposis (FAP) patients with molecularly-identified germline *APC* mutation (respectively Glu1309fsX1312 and Ser843fsX860). Collection and analysis of samples and clinico-pathological data were approved by the *G. D'Annunzio* University Ethical Committee and by the Institutional Review Board of the DDRC, Shariati Hospital, University of Tehran (protocol dated 17/03/2004). All cases were anonymized.

### IHC

TMA and standard whole tissue sections were cut at 4 µm and stained with anti-IRS1 rabbit polyclonal (C-20, sc-559, Santa Cruz Biotechnology, Heidelberg, Germany) at 1∶300 dilution for 30 min, after antigen retrieval by microwave treatment at 750 W for 10 min in 10 mM sodium citrate buffer pH 6.0 (Dako, Glostrup, Denmark). The anti-rabbit EnVision kit (K4003, Dako) was used for signal amplification. Serial TMA sections were also incubated with the following mouse monoclonal antibodies: anti-ß-catenin (17C2, Novocastra Laboratories Ltd, Newcastle, UK), anti-p53 (DO7, Novocastra) and anti-Ki67 (MIB-1, Dako), for which antigen retrieval was performed by thermostatic bath at 96°C for 40 min in sodium citrate buffer (Dako), and anti-EGFR pharmDx (2-18C9, Dako), according to manufacturer's instructions. All immunoreactions were revealed by a streptavidin-biotin-enhanced peroxidase system (Super Sensitive Link-Label IHC Detection System, BioGenex, Milan, Italy). Positive and negative controls were included for each antibody and in each batch of staining.

For each marker the percentages of positive cells were estimated in four fields at 400× magnification (≈1000 cells). IRS1, Ki67, p53, EGFR and ß-catenin were considered positive when >1% of the tumor cells were stained, ß-catenin was scored separately for immunostaining in the cytoplasm, nucleus and along the cell membrane. The independent samples t-test was used to evaluate differences in IRS1 expression (% of positive cells) according to pathological and mutational features. Expression of IRS1 was correlated to that of each of the other markers by Spearman's rho test. The SPSS (version 15.0) program (SPSS Inc., Chicago, IL, USA) was used for all statistical analyses. All cited *P* values are two-sided; *P*<0.05 was considered as statistically significant.

The density of IRS1 immunostaining in paired cancer-uninvolved colonic epithelium, primary CRC and liver metastasis was determined by semiquantitative digital analysis using ImageJ software (http://rsbweb.nih.gov/ij/). Images were acquired at standardized bright-field settings (400× magnification). Captured color jpeg images were converted to greyscale 8 bit images, then the Region Of Interest manager function was used to outline cytoplasmic areas, each comprising 5–15 cells. Unwanted nuclear or stromal elements were edited out. The areas analyzed in 4 matched sets of crypt epithelium, primary CRC and liver metastasis were 11 for bottom crypt epithelium, 10 for top crypt epithelium, 78 for total colonic epithelium, 84 for primary CRC, and 84 for metastatic CRC. Using default ImageJ settings, the units measured for each area were density value, total area in square pixels, average size and area fraction. Density values for crypt epithelium, primary CRC and metastatic CRC, normalized per area sizes, were analysed using unpaired Student's t-test. A value of *P*<0.05 was considered statistically significant [26].

### Quantitative real-time PCR (RTqPCR)

Total RNA from paired mucosa and CRC samples was isolated with QIAzol (QIAGEN, Hilden, Germany), treated with DNAase-1 (Ambion), checked by spectrophotometry and agarose gel electrophoresis, and retro-transcribed with the High Capacity DNA Archive Kit (Applied Biosystems), following manufacturer's instructions. RTqPCR assays were performed in duplicate using 96-well optical reaction plates and an ABI 7500HT machine (Applied Biosystems). Baseline amplification plot values were set automatically and thresholds were kept constant to obtain normalized cycle times and linear regression data. The reaction mix per well contained 10 µl Power Syber Green (Applied Biosystems), 2.4 µl of primers (final concentration 150 nM), 4.6 µl RNAase-free water, 3 µl cDNA (60 ng). For all experiments the PCR protocol was: denaturation at 95°C for 10 min, then 40 cycles at 95°C for 15 sec and at 60°C for 60 sec. Quantification was performed relative to *Cyclophilin* [27] using the ΔΔCT method. Validated RTqPCR primers, designed with Primer Express 3.0 software, were: *Cyclophilin*, FW 5′TTTCATCTGCACTGCCAAGA3′; RV5′TTGCAAAACACCACATGCT3′; *IRS1*, FW 5′GCAACCAGAGTGCCAAAGTGA3′ RV5′GGAGAAAGTCTCGGAGCTATGC3′; *c-MYC*, FW5′CCACCACCAGCAGCGACT3′, RV5′CAGAAACAACATCGATTTCTTCCTC3′.

### Cell cultures

Modulation of IRS1 and of the insulin/IGF1 axis was investigated in the Caco-2 and HT29 CRC cell lines, which, under specific culture conditions, undergo spontaneous *in vitro* differentiation [22], [28], [29]. Caco-2, developed from a primary CRC excised from a 72 yrs old male Caucasian, is MSI-stable and carries an inactivating *APC* point mutation, with second hit by loss of heterozygosity (LOH), a missense mutation in *ß-catenin* exon 5 (which does not appear to affect degradation), and is wild-type for *KRAS*, *BRAF*, *PIK3CA* and *PTEN* [23], [30]–[32]. HT29, developed from a primary CRC excised from a 44 yrs old female Caucasian, is also MSI-stable and carries double-hit inactivating *APC* mutations (which still allow limited ß-catenin phosphorylation and ubiquitination), as well as mutations in *SMAD4*, *BRAF*, *TP53*, and *PI3KCA*, encoding the p110α catalytic subunit of the class I phosphatidylinositol 3-kinases (PI3K) [23], [31] (see also the Catalogue of Somatic Mutations in Cancer, http://www.sanger.ac.uk/cosmic).

Caco-2 and HT29 cells were obtained from ATCC (ATCC-LGC Promochem, London UK). Caco-2 cells were maintained in Dulbecco's modified Eagle's medium (DMEM) with 10% fetal bovine serum (FBS), L-glutamine (2 mM), penicillin (100 units/ml), and streptomycin (100 µg/ml) under a humidified atmosphere with 5% CO2 at 37°C. Cells were plated on 10 cm Petri dishes and allowed to grow at confluency. Upon confluency (designated as zero time point) Caco-2 culture was carried out in DMEM with 20% FBS. The entire time course was performed twice and whole cell lysates were obtained at 3, 7 and 14 days from confluency. HT29 cells were maintained in DMEM with 10% FBS and allowed to grow for 3 (preconfluent), 7 (confluent) and 14 (post-confluent) days, at which times whole cell lysates were obtained.

### Western blotting

Whole cell or whole tissue lysates were prepared using ice-cold lysis buffer (100 mM NaCl, 10 mM EDTA, 1% TritonX-100, 50 mM Hepes pH 7.9, 10 mM NaF, 4 mM sodium pyrophosphate, 2 mM Na3VO4) supplemented with protease inhibitors (1 mM phenylmethylsulphonylfluoride, 2 µg/ml aprotinin, 2 µg/ml leupeptin). Lysates were cleared by centrifugation (10000× g for 20 min) and protein content was determined by the Bradford method. Fifty micrograms (50 µg) of total proteins were resolved under reducing conditions on 7.5% SDS-PAGE and transferred to reinforced nitrocellulose. The membrane was blocked with 3% not-fat dry milk in PBS with 0.01% Tween 20 for 1 hr at room temperature and then incubated overnight with the following primary antibodies: anti-IRS1 rabbit polyclonal (C-20, Santa Cruz), diluted 1∶500; anti-tyrosine 632-phosphorylated IRS1 (pIRS1 Tyr632) polyclonal antibody (Santa Cruz), diluted 1∶200; anti-ß-catenin monoclonal (Ylem, Rome, Italy), diluted 1∶50 or anti-ß-catenin polyclonal (#9562, Cell Signaling, Danvers, MA, USA), diluted 1∶1000; polyclonal against the ß subunit of the InsR (InsRß) (C-19, Santa Cruz) diluted 1∶200; polyclonal against the ß subunit of the IGF1R (anti-IGF1Rß, Cell Signaling Technology/Euroclone, Milan, Italy) diluted 1∶800; anti-actin monoclonal (Sigma-Aldrich, Milan, Italy) diluted 1∶10000. The membrane was then washed in PBS and incubated for 1 h at room temperature with the corresponding horseradish peroxidase-conjugated secondary antibody, diluted 1∶2000 (Ge Healthcare, Milan, Italy). Bound antibodies were detected using the enhanced chemiluminescent (ECL) method (Pierce-Celbio, Pero, Italy). Quantification of western blot signals (mean ± SE from at least two independent experiments) was obtained analyzing digitized signals with ImageJ software (http://rsbweb.nih.gov/ij/). The data were normalized for ß-actin and expressed as percentage of the maximum value.

### Immunofluorescence

Caco-2 and HT29 cells, grown on coverslips under the conditions described above, were fixed in methanol (−20°C) and incubated with anti-ß-catenin monoclonal (BD Science, Franklin Lakes, NJ, USA), anti-IRS1 rabbit polyclonal (C-20, Santa Cruz), diluted 1∶50; and anti-pIRS1 (Tyr632) polyclonal (Santa Cruz), diluted 1∶50. Nuclei were stained with 4,6-diamido-2-phenylindole (DAPI, Sigma-Aldrich) and cell membranes with wheat germ agglutinin (WGA, Sigma-Aldrich). Primary antibodies were visualized using goat anti-mouse IgG fluorescein isothiocyanate-conjugated (Cappel, MP Biomedicals Europe, Illkirch, France) or goat anti-rabbit IgG-Texas-Red-conjugated (Jackson ImmunoResearch Laboratories Europe, Newmarket, Suffolk, UK) for 30 min at room temperature. Cells were analyzed using an Apotome Axio Observer Z1 inverted microscope (Zeiss, Oberkochen, Germany) equipped with an AxioCam MRM Rev.3 at 40× magnification. Colocalization of the fluorescence signals was analyzed with AxioVision 4.6.3 software. Image analysis was performed using Adobe Photoshop.

### Electron microscopy

Cells were fixed in a mixture of 2% paraformaldehyde-2% glutaraldehyde in PBS (pH 7.4), post-fixed in 1% osmium tetroxide in veronal acetate buffer (pH 7.4) for 1 h at 25°C, stained with 0.1% tannic acid in the same buffer for 30 min at 25°C and with uranyl acetate (5 mg/ml) for 1 h at 25°C, dehydrated in acetone and embedded in Epon 812. Thin sections were finally examined under a Philips CM10 transmission electron microscope, after post-staining with uranyl acetate and lead citrate.

## Results

### IRS1 in CRC

**Figure 1:**
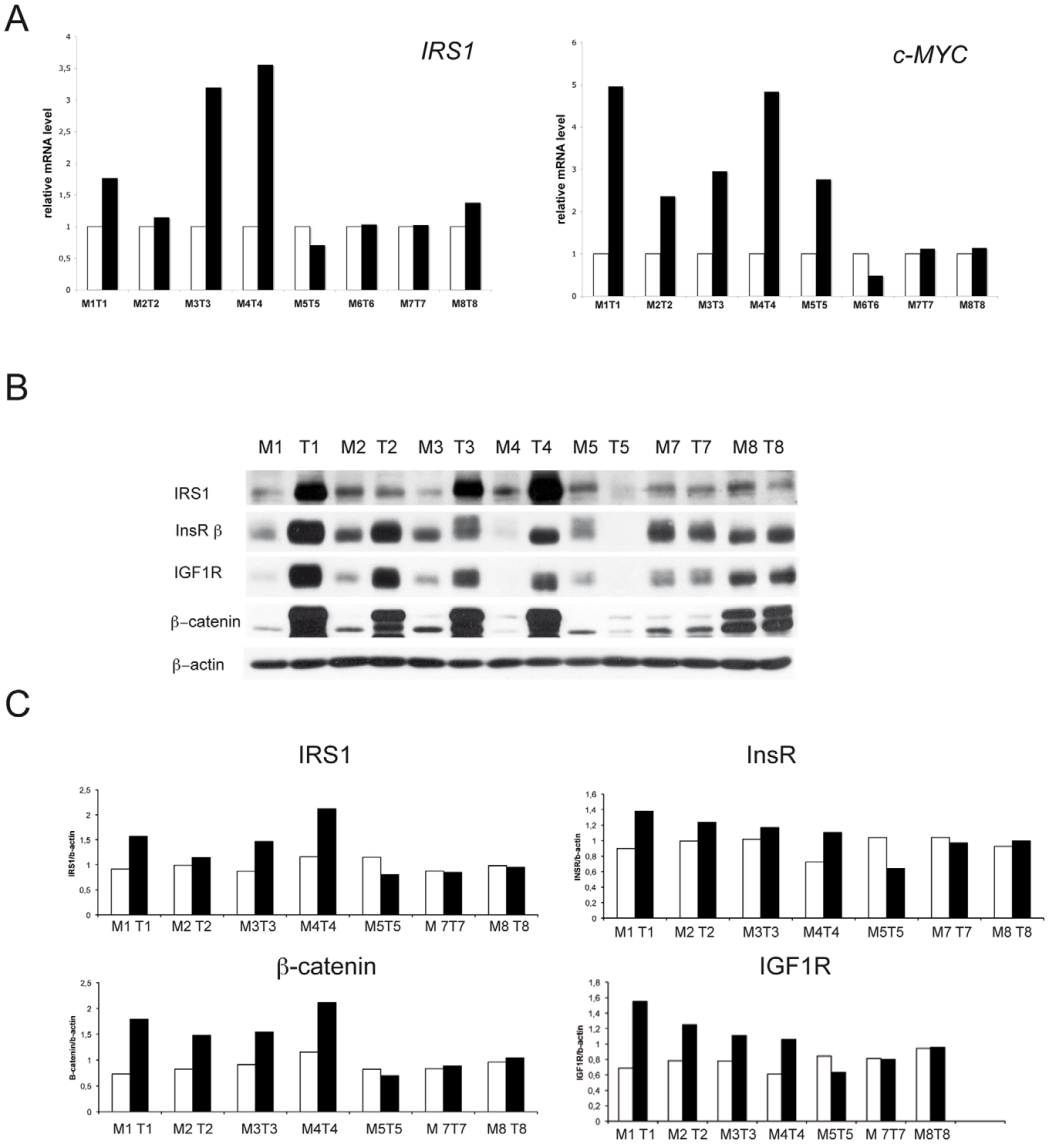
mRNA and protein levels of IRS1, *c-MYC*, insRß, IGF1R and ß-catenin in paired colonic mucosa and primary colorectal cancer (CRC). Panel A shows histograms of the relative expression of *IRS1* (left) and *c-MYC* (right) transcripts in paired samples of cancer-unaffected colorectal mucosa (white) and CRC (black), as determined by quantitative real-time PCR (RTqPCR). Mucosa samples were set equal to 100% and normalized to the relative expression of the housekeeping gene, *Cyclophilin*. Cancer samples were expressed relative to mucosa and normalized to the relative expression of the housekeeping gene. In pairs M1T1 to M4T4 and in M8T8, both *c-MYC* and *IRS1* increase in CRC relative to mucosa, only in M5T5 and M6T6 *IRS1* and *c-MYC* disagree (*IRS1*: *P* = 0.05, *c-MYC*: *P*<0.001, unpaired t test on the means of all differences, data not shown). Panel B shows western blot analysis of IRS1, beta subunit of the insulin receptor (InsRß), beta subunit of the insulin-like growth factor 1 receptor (IGF1Rß), ß-catenin and ß-actin, as loading control, in the paired colonic mucosa and CRC samples shown in A (except M6T6, for which tissue for western blot analysis was not available). The histograms in Panel C show quantitations, after normalization for ß-actin, of the IRS1, InsRß, IGF1Rß and ß-catenin signals. Relative to paired mucosa, IRS1 is overexpressed in the CRCs of pairs M1T1-M4T4, together with InsRß, IGF1Rß and ß-catenin (IRS1: *P* = 0.017, InsRß: *P* = 0.044, IGF1Rß: *P*<0.001, ß-catenin: *P*<0.001, unpaired t test on the means of all differences, data not shown). Notably, the CRCs that overexpressed the IRS1, InsRß, IGF1Rß and ß-catenin proteins also overexpressed *IRS1* and c-*MYC* mRNA.

We determined by RTqPCR the constitutive expression of *IRS1* and of *c-MYC,* key WNT target and effector [17], in total RNA from paired colorectal mucosa and CRC samples (Figure 1A). Five CRCs overexpressed *IRS1* relative to paired mucosa. Overall, the mRNA levels of *IRS1* were in good agreement with those of *c-MYC*.

To explore the modulation of IRS1 and of other insulin/IGF pathway components, we assessed by western blot the protein levels of IRS1, InsRß, IGF1Rß and ß-catenin in 7 of the 8 above-reported CRC cases (for which tissue was available), and in paired colonic mucosa and adenoma samples from two unrelated FAP patients [33]. In the primary CRCs the IRS1 protein levels reflected the mRNA levels, being higher, relative to paired mucosa, in 4 of the 5 cases that overexpressed *IRS1* and *c-MYC* mRNA. These CRCs also overexpressed InsRß, IGF1R, and ß-catenin, while in the other cases the mucosal levels of InsRß, IGF1R, and ß-catenin were similar or above those of the paired CRC (Figure 1B–C). In the two FAP cases, IRS1 markedly increased in the adenoma relative to mucosa, together with InsRß, IGF1Rß and ß-catenin, and IHC showed diffuse IRS1 in adenomas (Figure S1).

**Figure 2:**
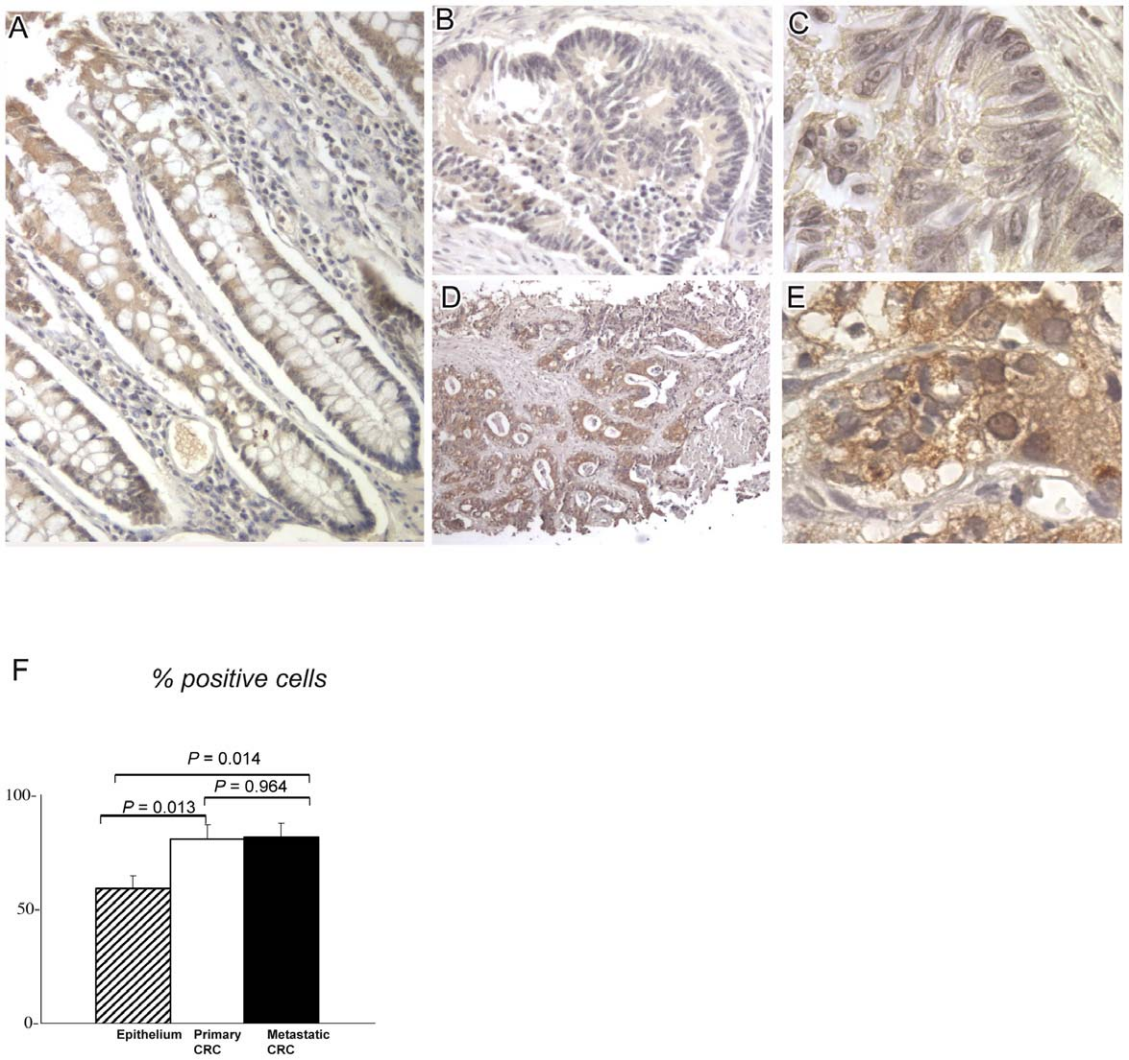
IRS1 immunostaining in cancer-uninvolved colonic epithelium, primary colorectal cancer and paired synchronous liver metastases. Panel A shows IRS1 immunostaining in full-length longitudinal sections of cancer-uninvolved colonic crypts. Panels B–E provide an example of IRS1 immunostaining in primary CRC (B–C) versus paired metastasis (liver biopsy core, D–E). Both show diffuse cytoplasmic IRS1, with much stronger immunostaining in metastatic cells. Panel F shows the histograms of the mean percentages of IRS1-positive cells in 24 cases of matching non-neoplastic colon epithelium, primary CRC and metastatic CRC (error bars mean ± SEM). There were significant differences between colonic epithelium (59.1±5.6%) and primary CRC (80.8±6.2%, *P* = 0.013 by independent sample t test) and between epithelium (59.1±5.6%) and hepatic metastasis (81.3±6.6%, *P* = 0.014). The difference between primary and metastatic CRC was not significant (*P* = 0.964).

**Figure 3:**
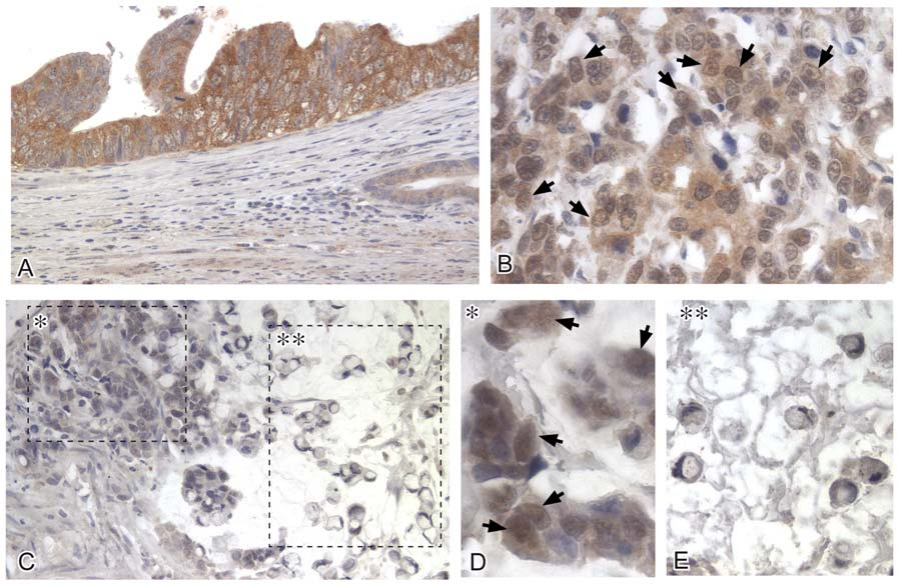
IRS1 and tumor histotype in primary colorectal cancer. Panels A and B respectively show diffuse cytoplasmic IRS1 in non-mucinous colorectal CRCs, including a moderately differentiated tumor, with strong immunostaining of cancer cells, and a poorly differentiated tumor, with weaker and possibly also nuclear IRS1 (arrowheads). Panels C–E show a poorly differentiated CRC with mucinous, mostly signet-ring phenotype. Notably, in the marginal area (single asterisk) detailed in panel D, tumor cells with non-mucinous phenotype show nuclear/perinuclear IRS1 (arrowheads), whereas signet-ring cells floating in mucin (double asterisk), detailed in panel E, do not show IRS1 immunostaining.

We further assessed IRS1 protein expression by IHC in individually-matched paraffin-embedded sections of colonic mucosa, primary and metastatic CRC. Twenty-four cases with paired primary CRC and liver metastasis, also including cancer-uninvolved colonic mucosa, were available for analysis. Immunoreactive IRS1 was clearly detectable in crypt epithelium, as well as in primary and metastatic CRC (Figure 2A–E). Primary and metastatic tumors, when compared to colonic epithelium, contained higher numbers of cells expressing IRS1 (80.8±6.2% for primary and 81.3±6.6% for metastatic CRC versus 59.1±5.6% for colonic epithelium, *P* = 0.013 and *P* = 0.014, respectively, Figure 2F). Density values of pixels for IRS1, as quantified using ImageJ software, did not differ between primary CRC and colonic epithelium, but were significantly higher in liver metastases compared to CRC (*P*<0.01) and colonic epithelium (*P*<0.01) (Table 1). Differences in density values of IRS1 between colonic epithelium of bottom and top crypt were not significant. We further explored the pathologic correlations of IRS1 expression in a series of 163 primary CRCs tested by IHC on TMA (Table 2). Overall 151/163 cases (92.6%) were IRS1-positive. IRS1 expression did not significantly differ in relation to age at diagnosis, gender, tumor location (right versus left colon), Duke's stage, and MSI status. However, CRCs with high/moderate differentiation were more likely to show high percentages of IRS1-positive cells than poorly-differentiated tumors (*P* = 0.001), while CRCs with mucinous/signet-ring phenotype were associated with focal or no IRS1 (*P*<0.001). Poorly differentiated CRCs often manifested nuclear staining in addition to cytoplasmic reactivity. Figure 3A–E exemplifies IRS1 staining patterns.

**Table 1:** Density of IRS1 immunostaining in paired colonic epithelium, primary colorectal cancer and synchronous liver metastasis.

Sample type	Mean ± SEM	*P*
Bottom crypt (11)*	39.10±2.9	
Top crypt (10)	45.40±2.16	*0.15*
Total colonic epithelium (78)	42.00±1.60	
Primary CRC (84)	44.50±0.99	*0.36*
Total colonic epithelium (78)	42.00±1.60	
Liver metastasis(84)	70.38±2.6	***<0.01***
Primary CRC (84)	44.50±0.99	
Liver metastasis(84)	70.38±2.60	***<0.01***

Density values (mean ± SEM), normalized per area size in square pixels, were obtained by digital analysis using ImageJ software (http://rsbweb.nih.gov/ij/) for IRS1 staining in: bottom versus top colonic crypt epithelium (respectively 11 and 10 areas); total colonic epithelium (78 areas) versus primary CRC (84 areas); total colonic epithelium (78 areas) versus liver metastasis (84 areas); primary CRC versus liver metastasis (84 areas). Only the differences between total colonic epithelium and liver metastasis and between primary CRC and liver metastasis are significant (*P*<0.01).

* Number of examined areas, CRC: colorectal cancer.

**Table 2:** Independent samples t-test of IRS1 expression according to the individual and pathological features of the primary colorectal cancer cases (n = 163).

		% IRS1 positive cells	
Variable	n (%)	mean ± SE	*P*
**Age at diagnosis (yr)**			
≤40	60 (36.8)	79.0±4.2	0.144
>40	103 (63.2)	86.1±2.4	
**Gender**			
Male	90 (55.2)	83.2±2.9	0.877
Female	73 (44.8)	83.9±3.3	
**Tumor location**			
Right colon	58 (35.6)	78.6±4.3	0.168
Left colon/rectum	105 (64.4)	85.6±2.5	
**Differentiation**			
Well+Moderate	138 (84.7)	88.1±1.9	**0.001**
Poor	25 (15.3)	58.0±8.2	
**Duke's stage**			
A+B	91 (55.8)	85.7±2.8	0.252
C+D	72 (44.2)	80.7±3.5	
**Mucinous** *			
Yes	42 (25.8)	67.1±6.1	**0.001**
No	121 (74.2)	89.2±1.8	
**MSI**			
Absent	124 (76.1)	84.3±2.5	0.536
Present	39 (23.9)	81.2±4.7	
***KRAS*** †			
Wild-type	98 (65.8)	82.0±3.0	0.442
Mutated	51 (34.2)	85.8±3.4	

* comprising mucinous and signet-ring carcinomas;

† *KRAS* mutations data not available for 14 cases. Significant correlations (p<0.05) in bold.

As shown in Table 3, which reports Spearman's correlations between IHC markers, IRS1 was positively associated with Ki67 (*P* = 0.008), p53 (*P* = 0.032), membrane (*P* = 0.001) and cytoplasmic (*P*<0.001) ß-catenin. Other associations involved cell membrane ß-catenin, positively correlated with cytoplasmic ß-catenin (*P*<0.001) and EGFR (*P* = 0.005), and cytoplasmic ß-catenin, positively correlated with nuclear ß-catenin (*P*<0.001), Ki67 (*P* = 0.002) and p53 (*P* = 0.009), while nuclear ß-catenin positively correlated with p53 (*P* = 0.049). Predictably, a positive correlation was found between Ki67 and p53 (*P* = 0.029).

**Table 3:** Spearman's correlations among IRS1, Ki67, p53, EGFR and ß-catenin in the primary colorectal cancer cases (n = 163).

	IRS-1	Ki-67	p53	EGFR	ßCat M	ßCat C	ßCat N
**IRS1**							
Rho	1	**0.213**	**0.168**	−0.030	**0.263**	**0.360**	0.128
*P*		**0.008**	**0.032**	0.709	**0.001**	**0.000**	0.107
**Ki67**							
Rho	**0.213**	1	**0.176**	−0.049	0.082	**0.251**	0.074
*P*	**0.008**		**0.029**	0.554	0.311	**0.002**	0.361
**p53**							
Rho	**0.168**	**0.176**	1	−0.088	0.108	**0.205**	**0.156**
*P*	**0.032**	**0.029**		0.277	0.175	**0.009**	**0.049**
**EGFR**							
Rho	−0.030	−0.049	−0.088	1	**0.222**	0.037	0.156
*P*	0.709	0.554	0.277		**0.005**	0.645	0.050
**ßCat M**							
Rho	**0.263**	0.082	0.108	**0.222**	1	**0.509**	−0.088
*P*	**0.001**	0.311	0.175	**0.005**		**0.000**	0.267
**ßCat C**							
Rho	**0.360**	**0.251**	**0.205**	0.037	**0.509**	1	**0.491**
*P*	**0.000**	**0.002**	**0.009**	0.645	**0.000**		**0.000**
**ßCat N**							
Rho	0.128	0.074	**0.156**	−0.157	−0.088	**0.491**	1
*P*	0.107	0.361	**0.049**	0.050	0.267	**0.000**	

Abbreviations: ßCat: ß-catenin; EGFR: epidermal growth factor receptor; M: membrane staining; C: cytoplasmic staining; N: nuclear staining; Rho: Spearman's coefficient correlation. Significant correlations (*P*<0.05) in bold.

These results suggest that high IRS1 is associated with CRCs combining well/moderately differentiated histological phenotype with immunohistochemical markers of poor prognosis (expression of Ki67, p53, and cytoplasmic ß-catenin).

### IRS1 in Caco-2 polarization

**Figure 4:**
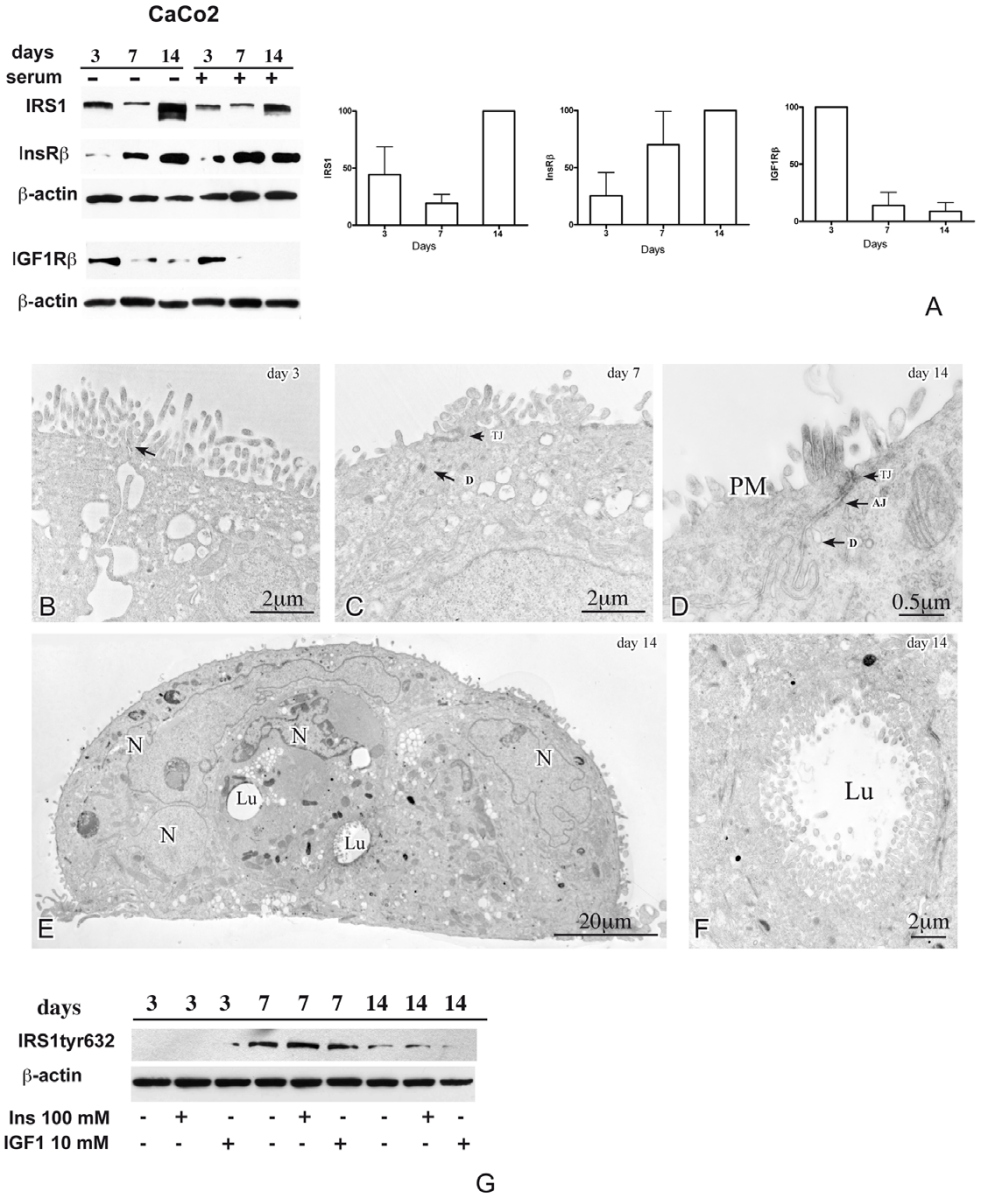
Expression of IRS1, insulin receptor, IGF1 receptor and ultrastructural differentiation in polarizing Caco-2 cells. Panel A shows western blot analysis of IRS1, beta subunit of the insulin receptor (InsRß), beta subunit of the insulin-like growth factor 1 receptor (IGF1Rß) and ß-actin, as loading control, in Caco-2 cells at days 3, 7 and 14 post-confluence, duplicated in absence (−) and presence (+) of serum in the culture medium. The histograms show quantitations, after normalization for ß-actin, of the IRS1, InsRß and IGF1Rß signals (means ± SE from the two experiments). Under both culture conditions increased espression of IRS1 and InsRß is clearly evident in polarized cells at day 14 (IRS1) and at days 7 and 14 (InsRß), whereas maximum expression of IGF1Rß is detected only at day 3. Transmission electron microscopy of Caco-2 cells at day 3 of the spontaneous polarization time course reveals forming electron-dense junctions at the apex of the lateral membranes of adjacent cells (panel A, arrow). With progression of polarization, tight junctions and desmosomes (panels C–D, arrows) and adhesion junctions (panel D) become evident as electron-dense plaques on adjacent lateral membranes at days 7 and 14, respectively. In addition, tight multicellular clusters, with differentiation features, such as intracellular lumina rich of apical brush border (panels E–F), become evident at day 14. Abbreviations: tj, tight junction; ad, adhesion junction; ds, desmosome. Panel G shows western blot levels of tyrosine 632-phosphorylated IRS1 (IRS1tyr632) and, as loading control, ß-actin, in serum-starved Caco-2 cells unstimulated (−) and stimulated (+) with insulin (100 nM) or IGF1 (10 nM). IRS1 tyrosine phosphorylation is relevant at day 7 of polarization, independently from the addition of exogenous insulin or IGF1. However, at day 3, only exogenous IGF1 determines IRS1 phosphorylation.

Caco-2 carries an inactivating APC mutation with second hit by LOH, but is known to be negative for mutations in *KRAS*, *BRAF*, *PIK3CA* and *PTEN* [23], [31] (see also COSMIC, http://www.sanger.ac.uk/cosmic). Caco-2 cells are capable of spontaneous differentiation, documented by the expression of microvilli, enzymes and transporters characteristic of polarized enterocytes and by the development of tight junctions, which, in the *in vivo* setting, are necessary for upward migration of crypt epithelial cells toward the mucosal surface [28], [29], [34]. IRS1 expression and activation was analyzed by western blot in Caco-2 cultures during polarization at 3, 7 and 14 days after confluency, in presence and in absence of serum, together with expression of InsRß and IGF1Rß (Figure 4A). Under both culture conditions IRS1 decreased at day 7, but increased subsequently, with highest expression at day 14. Notably, in both normal and serum-free cultures, InsRß resulted weakly expressed at day 3 and significantly increased at day 7, with maximum expression at day 14. Inversely, again under both conditions, IGF1Rß was highest at day 3 and dramatically decreased at days 7 and 14. To assess IRS1 activation, Caco-2 cells at 3, 7 and 14 days from confluency were serum-starved overnight and then stimulated with or without insulin (100 nM) or IGF1 (10 nM) for 5 min. Total protein lysates (80 µg) obtained after 5 min of treatment were resolved and blotted with anti-pIRS1 Tyr632 (Figure 4G). IRS1 tyrosine phosphorylation was relevant at day 7 of polarization and did not appear to be modulated by exogenous insulin or IGF1 (lane 4–6). However, at day 3, only exogenous IGF1 activated IRS1. These data suggest that IRS1 could mediate autocrinely-activated InsR signaling in polarized cells (where IRS1 and InsRß are maximally expressed and IGF1Rß is lowest), and IGF1R signaling, activated by exogenous IGFs, in pre-polarized cells (where IRS1 is expressed at lower level and IGF1Rß and InsRß at highest and lowest levels, respectively).

Transmission electron microscopy of the Caco-2 cultures documented at day 3 the formation of localized electron-dense areas of close opposition between adjacent lateral plasma membranes, characteristic of forming tight junctions, and at day 14 the presence of complete intercellular junctional complexes, as well as polarization of the absorptive apical brush border (Figure 4B–F). Overall, this confirmed enterocytic polarization during the culture time course [28].

**Figure 5:**
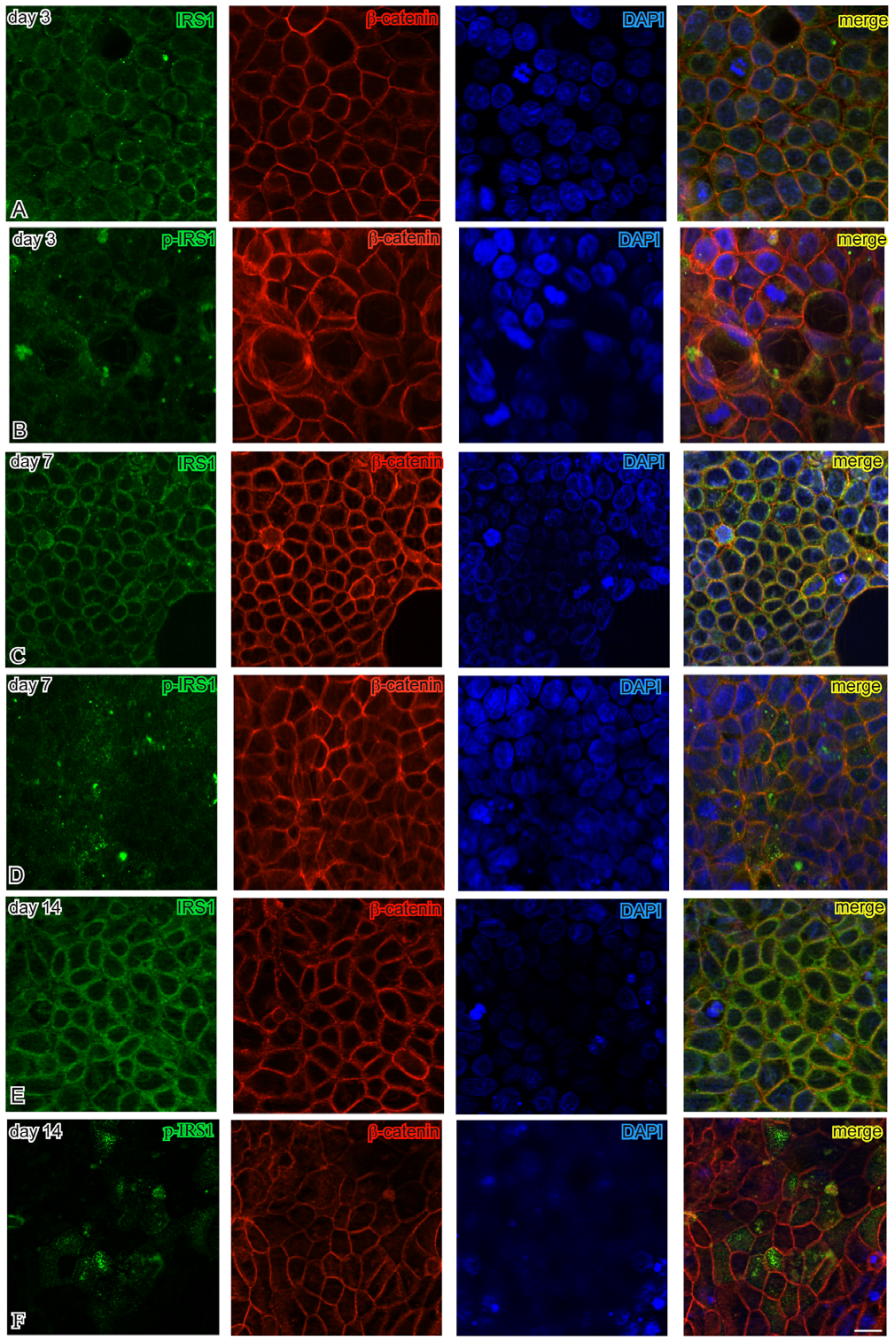
Subcellular localizations of IRS1, p-IRS1 and ß-catenin during Caco-2 polarization. Apotome immunofluorescence analysis at days 3, 7, and 14 postconfluency demonstrates differences in the cellular distribution of total IRS1 (IRS1, green), tyrosine 632-phosphorylated IRS1 (pIRS1, green), and ß-catenin (red) during the Caco-2 culture time course. For each field, the nuclei are counterstained in blue with 4′,6-diamidino-2-phenylindole (DAPI). Overlaps between red and green signals (merge) point to co-localizations (in yellow) of IRS1/pIRS1 and ß-catenin. Bar = 20 µm.

Immunofluorescence analysis demonstrated differences in the subcellular distribution of total IRS1 and of pIRS1 Tyr632 during the Caco-2 culture time course (Figure 5). At day 3 IRS1 immunolabeling was distinctly less intense than at days 7 and 14, and was predominantly localized along the lateral and basolateral cell membranes. Merging of the ß-catenin and IRS1 images confirmed the colocalization of the two proteins along the basolateral membranes. At day 3 staining of pIRS1 Tyr632 was relatively weak and had a distribution similar to that of total IRS1. At day 7 pIRS1 Tyr632 increased and mostly appeared as punctate staining on top of surface cells, suggestive of localization beneath the plasma membrane on the apical side. Total IRS1 remained predominantly localized along the basolateral membranes, together with ß-catenin. Notably, at day 14 pIRS1 Tyr632 was restricted to fewer surface cells, which, however, appeared more strongly labeled than at day 7, with the typical apical plasma membrane punctate pattern (the lower number of positive cells was consistent with the decrease in pIRS1 Tyr632 at day 14 by western blot). No nuclear staining for IRS1, pIRS1 Tyr632 or ß-catenin was observed during Caco-2 cell polarization.

In conclusion, the immunofluorescence data indicate that in the Caco-2 model the intensity of IRS1 and pIRS1 staining increases with polarization, and that, during such process, pIRS1 Tyr632 becomes expressed in surface cells only, switching in subcellular distribution from the cytoplasm and basolateral membranes, where it colocalizes with ß-catenin, to the apical plasma membranes. This parallels the tightening of the intercellular junctions, as evidenced by electron microscopy, which makes the basolateral membranes inaccessible to freely-diffusing extracellular molecules.

### IRS1 in HT29 polarization

HT-29 cells carry double-hit *APC* mutations, as well as mutations in *SMAD4*, *BRAF*, *TP53*, and, particularly, *PI3KCA*, which constitutively activate and deregulate intracellular signaling [23], [31] (see also COSMIC, http://www.sanger.ac.uk/cosmic). Therefore HT29 cells provide a distinct *in vitro* model for the study of intestinal epithelial differentiation and polarity [22], [29].

**Figure 6:**
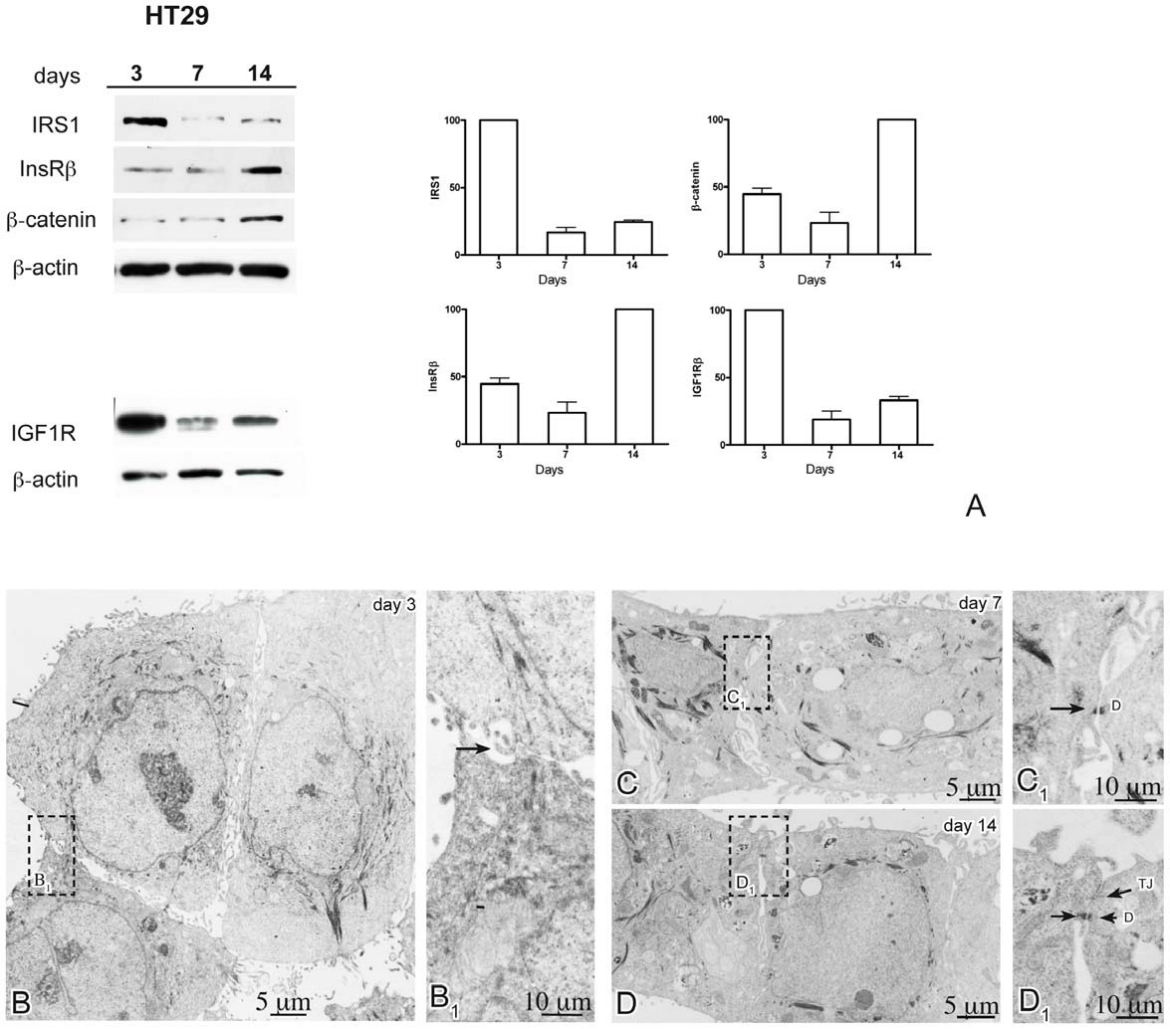
Expression levels of IRS1, insulin receptor, IGF1 receptor, ß-catenin and ultrastructural differentiation in polarizing HT29 cells. Panel A shows western blot analysis of IRS1, beta subunit of the insulin receptor (InsRß), ß-catenin, beta subunit of the insulin-like growth factor 1 receptor (IGF1Rß), and ß-actin, as loading control, in HT29 cells maintained in complete medium during spontaneous differentiation at days 3 (pre-confluent), 7 (confluent) and 14 (post-confluent). The histograms show quantitations, after normalization for ß-actin, of the IRS1, InsRß, ß-catenin and IGF1Rß protein signals (means ± SE from two independent experiments). Expression of IRS1 and IGF1Rß is highest at day 3 and markedly declines at days 7 and 14, whereas InsRß is maximally expressed at day 14. At day 3, transmission electron microscopy of HT29 cells reveals bundles of intermediated filaments converging towards the plasma membrane to form electron-dense junctions between adjacent cell membranes (panel B–B1, arrow). With progression of the time-course, HT29 cells display differentiated features, such as desmosomes at days 7 and 14 (panel C–C1, arrow, and D–D1, arrow) and tight junctions at day 14 (D–D1, arrow). Abbreviations: tj, tight junction; ad, adhesion junction; ds, desmosome.

IRS1 protein expression was analyzed by western blotting in HT29 cultures during spontaneous polarization, together with the InsRß and IGF1Rß subunit proteins. Western blot analysis showed that IRS1 levels were highest at day 3 (pre-confluent) and markedly decreased at day 7 (confluent), with only a slight increase (relative to day 7) at day 14 (post-confluent) (Figure 6A). IGF1Rß was similarly modulated, showing highest expression at day 3, marked decrease at day 7, and only slight increase (relative to day 7) at day 14. InsRß, low at day 3 and lowest at day 7, strongly increased at day 14. ß-catenin demonstrated little modulation, with slightly higher level at day 14 (post-confluent).

Transmission electron microscopy analysis of pre-confluent (day 3), confluent (day 7) and post-confluent (day 14) HT29 cells demonstrated gradual polarization during the time course (Figure 6B–D). Formation of localized electron-dense areas of close opposition between the lateral plasma membranes of adjacent cells, characteristic of forming intercellular junctions, was evident at day 7, and tight junctions, including zonula adherens and desmosomes, were evident at day 14, together with functional apical polarization of the microvilli.

**Figure 7:**
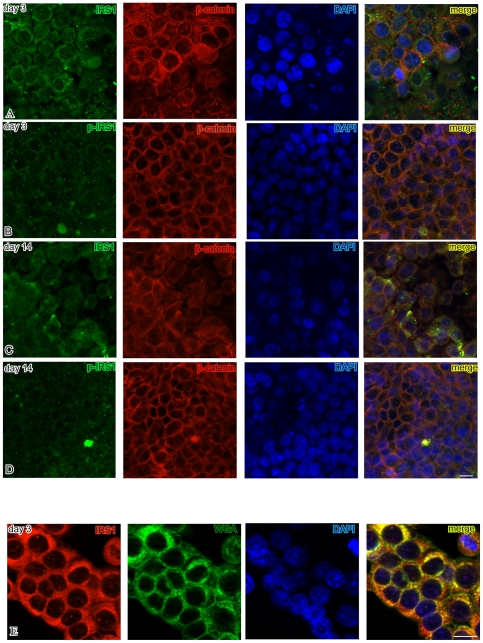
Subcellular localizations of IRS1, p-IRS1 and ß-catenin during HT29 polarization. Panels A–D show Apotome immunofluorescence analysis of HT29 cells at days 3 (pre-confluent), 7 (confluent) and 14 (post-confluent). This demonstrates differences in the cellular distribution of total IRS1 (IRS1, green), tyrosine 632-phosphorylated IRS1 (pIRS1, green, mainly nuclear dots), and ß-catenin (red) during the culture time course. For each field, the nuclei are counterstained in blue with 4′,6-diamidino-2-phenylindole (DAPI). Overlaps between red and green signals (yellow) point to co-localizations of IRS1/pIRS1 and ß-catenin. This does not appear to involve the nuclear localizations, where IRS1 mainly appears as green dots and ß-catenin mainly as purple to bluish-violet blotches. Panel E highlights nuclear IRS1 (red, discrete dots) by double labeling of HT29 cells with the membrane marker wheat germ agglutinin (WGA, green). Bars = 20 µm.

Immunofluorescence analysis clearly revealed that in pre-confluent, and, with lesser intensity, post-confluent HT29 cells IRS1 was detectable in the cytoplasm, particularly in the perinuclear region, as well as in discrete spots within the nuclei (Figure 7). Nuclear IRS1 was confirmed by double labeling with IRS1 and the membrane marker WGA. Interestingly, pIRS1 Tyr632, which appeared as discrete dots, was almost exclusively localized in the nuclei and did not change between pre- and post-confluent cells. At both culture time points, ß-catenin was present in the nucleus, as well as in the cytoplasm and along the lateral membranes. IRS1 did not colocalize with ß-catenin in pre-confluent cells, while some colocalization signals, only in the cytoplasm, were observed in post-confluent cells.

These data indicate that in HT29 cells, known to carry mutations that constitutively activate intracellular signaling, IRS1 is highest in pre-confluent cells and pIRS1 localizes in discrete nuclear spots, with little variation during the polarization course.

## Discussion

Of the 3 human IRS genes, IRS1, IRS2 and IRS4, the ubiquitously expressed IRS1 and IRS2 mediate the major metabolic, proliferative and anti-apoptotic functions of InsR and IGF1R signaling [18]. Tyrosine-phosphorylated IRS1/2 bind Src homology 2 (SH2) proteins, such as the p85 subunit of the PI3K, the phosphotyrosine phosphatase SHP-2, the Src-like kinases Fyn, Grb-2, NCK, CRK, SHB, and others. These activate downstream effectors, such as the mitogen-activated protein kinase (MAPK) and the PI3K pathways, which promote survival, proliferation, differentiation and metabolic responses [18].

We previously showed that IRS2 is directly controlled by the caudal-related homeobox protein 2 (CDX2) and significantly expressed in top crypt intestinal epithelium [27]. *IRS2* RNA increases with spontaneous differentiation in both HT29 and Caco-2 cells and is downregulated in ß-catenin-dependent intestinal tumorigenesis [33].

In contrast, several lines of evidence, summarized in the introduction, implicate IRS1 in intestinal carcinogenesis, under the direct control of TCF/LEF-ß-catenin complexes [19]–[21]. It is also well known that endogenous IRS1 is overexpressed and constitutively activated in a variety of human cancers [34].

In the present study, *IRS1* mRNA and protein levels resulted higher, relative to paired mucosa, in CRCs that overexpressed *c-MYC*, ß-catenin, InsRß, and IGF1R. Overexpression of IRS1, together with ß-catenin, InsRß, and IGF1R, in FAP-associated adenomas was in agreement with data reported for the *Apc(Min/+)* mouse model [21], [35]. By IHC, IRS1 resulted expressed throughout the colonic crypt, which could be consistent with a role of InsR/IGF1R signaling in intestinal epithelial differentiation [36]–[37].

The immunostaining of the primary CRCs was in the range of that of the cancer-uninvolved top crypt in terms of intensity, but more diffuse in terms of percentages of positive cells, which may account for the above-discussed higher mRNA and protein levels in CRC relative to mucosa. In the hepatic metastases, IRS1 positivity was similar to that of the primary tumors in percentages of stained cells, but the staining intensity resulted significantly higher. The increase of IRS1 staining intensity in hepatic metastases is consistent with the fact that the liver is the major site of IGFs synthesis [38]. Furthermore, it has been shown that metastatic CRC cells express high IGF1R and that IRS1 promotes liver metastatization [38]–[41].

Human CRC is a molecularly heterogeneous disease [42]–[45]. To investigate associations with pathological characteristics, we evaluated a series of 163 primary CRCs for IRS1 expression by TMA IHC. In terms of percentages of positive cells, IRS1 correlated with moderately/well-differentiated phenotype, but was also associated with markers of proliferative activity/biological aggressiveness (Ki67, p53, and cytoplasmic ß-catenin). In this regard, it is well recognized that the moderately to well-differentiated CRC subset includes tumors with a spectrum of histological variation and with different metastatic potentials [46]. Moreover, it has been shown that resistance to apoptosis identifies CRCs that, independently of clinicopathological variables (including grade of histological differentiation), have poor outcomes [47]. Signaling through the *IGF*/*INS*/IRS axis has a key anti-apoptotic role [48]. Further studies are needed to verify whether IRS1 concurs in identifying CRCs that, independently of classic pathological variables, have poor prognosis because of selective advantages during tumor progression [49].

Unlike Bommer et al. [19], who reported an enrichment of mucinous histotype in their high-IRS1 CRC subset, in our study mucinous/signet ring CRCs expressed significantly less IRS1 than non-mucinous tumors. In this regard, our findings are coherent with our evidence that IRS1 labeling correlates with moderately/well-differentiated phenotype and with the fact that mucinous/signet ring CRCs tend to be poorly differentiated [50].

To correlate IRS1 with differentiation in *in vitro* CRC models, we investigated the expression of IRS1, InsRß and IGF1R in Caco-2 and HT29 cells. In Caco-2, that mimics crypt to villus axis differentiation [28]–[29], IRS1 and InsRß reached maximum levels at completion of polarization, while IGF1Rß was maximally expressed in pre-polarized cells. This suggests that IRS1 could mainly mediate IGF1R signaling before polarization and InsR signaling with polarization, together with IRS2, also highly expressed in polarized Caco-2 cells [27], [51]. No nuclear IRS1 was detected, while, with polarization, pIRS1 Tyr632 was expressed only in surface cells, switching from the lateral to the apical plasma membrane. This process paralleled the tightening of the intercellular junctions evidenced by electron microscopy, suggesting that IRS1 migrated from intercellular membranes that, due to close-fitting contact, became inaccessible to exogenous signaling, to free apical membranes. Notably, this sub-apical localization is consistent with the fact that in polarized Caco-2 cells InsR and IGF1R are implicated in the regulation of Na+ glucose transport across the brush border, as in functional intestinal epithelium, and with evidence that in polarized epithelia insulin is in the apical fluid and InsR at the apical membrane [52]–[54]. Thus the Caco-2 model supports the view that cytoplasmic IRS1 expression correlates with differentiation, as observed by immunohistochemistry in primary CRCs.

In HT29 cells, where a gain of function *PI3KCA* mutation cooperates with other mutations in increasing proliferative and survival capacities [7], [43], [44], [55], total IRS1 decreased with polarization and pIRS1, that did not appreciably vary, localized mainly in discrete nuclear spots, in contrast with the sub-apical location seen in polarized Caco-2 cells. This nuclear localization could be relevant, as *IRS1* inhibition attenuates tumorigenicity in HT29 cells, that express low cytoplasmic IRS1 [19]. Following oncogene activation or IGF1 treatment, IRS1 is known to translocate in the nucleus [56]–[60], where it might be involved in ß-catenin translocation [61] and/or act as a transcription factor [57]. Furthermore, activated IGF1R has been recently shown to translocate to the nucleus in both non-malignant tissues and cancers [62]–[63], although it remains to be determined whether IRS1 and IGF1R interact in this subcellular location. Thus, the low IRS1 expression seen in HT29 cells could reflect that observed in the poorly differentiated primary CRCs, including mucinous/signet ring tumors, and could be related to nuclear trafficking and functions.

In conclusion, our results provide further evidence that IRS1 is differentially modulated, together with InsRß, IGF1R, and ß-catenin, during differentiation in the Caco-2 and HT29 cell models and *ex vivo* in primary CRCs versus mucosa. Furthermore, diffuse IRS1 appears to be associated with CRCs that, despite their moderately to well differentiated histologic features, express markers of biological aggressiveness. The increase in IRS1 immunostaining in hepatic secondaries is consistent with a possible role in liver metastatization [38]–[41]. Unfortunately, follow-up data for our patients were not available. Therefore, the prognostic value of IRS1 expression in CRC could not be assessed. This is a limitation of the present study, which needs to be addressed in future investigations.

## Supporting Information

Figure S1 **IRS1, insulin receptor, IGF1 receptor and ß-catenin in colonic mucosa and adenomas from familial adenomatous polyposis coli (FAP) patients.** Panel A compares the western blot expression levels of IRS1, beta subunit of the insulin receptor (InsRß), beta subunit of the insulin-like growth factor 1 receptor (IGF1Rß), ß-catenin and, as loading control, ß-actin, in paired mucosa (M) and adenoma (T) samples from two unrelated FAP patients. In both cases, the levels of IRS1, InsRß, IGF1Rß, and ß-catenin are distinctly higher in the adenoma versus the paired mucosa sample. Panel B, detailing the edge of an adenoma, highlights the difference in IRS1 immunostaining associated with the transition between normal-appearing (downward pointing arrow) and dysplastic (upward pointing arrow) colonic crypts. The hyperplastic and mucin-depleted epithelium of the dysplastic crypts shows diffuse cytoplasmic IRS1, while mostly perinuclear/nuclear IRS1 is evident in non-dysplastic crypts. Panel C shows diffuse cytoplasmic IRS1 in a severely dysplastic adenoma.
(TIF)
