# Supplementary figures and images for: The Insulin Receptor Substrate 1 (Irs1) in Intestinal Epithelial Differentiation and in Colorectal Cancer
Source: PLoS One. 2012 Apr 27;7(4):e36190. doi: 10.1371/journal.pone.0036190 (PMC3338610; doi:10.1371/journal.pone.0036190)

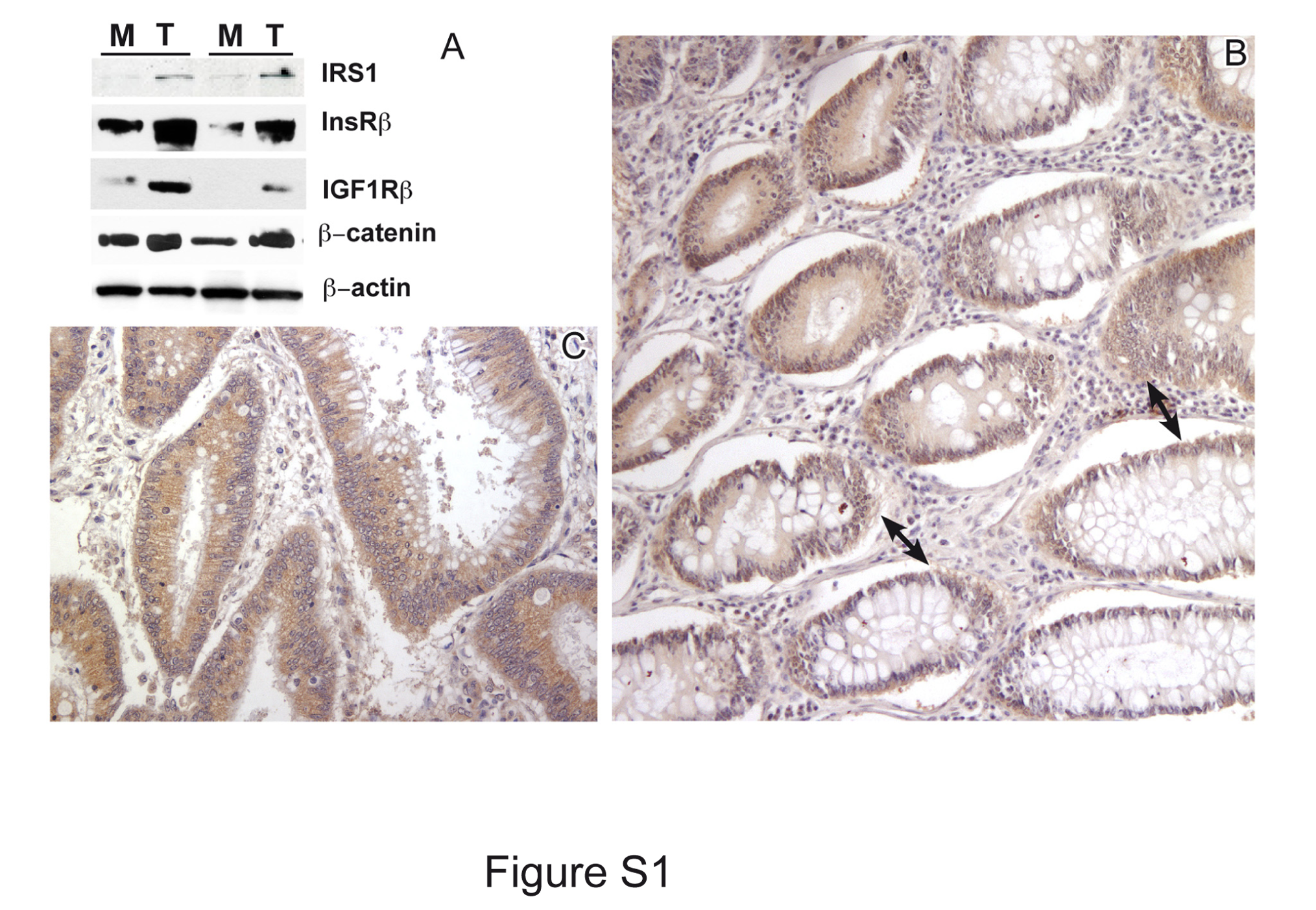

Supplement: Figure S1 — IRS1, insulin receptor, IGF1 receptor and ß-catenin in colonic mucosa and adenomas from familial adenomatous polyposis coli (FAP) patients. Panel A compares the western blot expression levels of IRS1, beta subunit of the insulin receptor (InsRß), beta subunit of the insulin-like growth factor 1 receptor (IGF1Rß), ß-catenin and, as loading control, ß-actin, in paired mucosa (M) and adenoma (T) samples from two unrelated FAP patients. In both cases, the levels of IRS1, InsRß, IGF1Rß, and ß-catenin are distinctly higher in the adenoma versus the paired mucosa sample. Panel B, detailing the edge of an adenoma, highlights the difference in IRS1 immunostaining associated with the transition between normal-appearing (downward pointing arrow) and dysplastic (upward pointing arrow) colonic crypts. The hyperplastic and mucin-depleted epithelium of the dysplastic crypts shows diffuse cytoplasmic IRS1, while mostly perinuclear/nuclear IRS1 is evident in non-dysplastic crypts. Panel C shows diffuse cytoplasmic IRS1 in a severely dysplastic adenoma. (TIF) [file pone.0036190.s001.tif]
